# The impact of polycystic ovary syndrome on attention: an empirical investigation

**DOI:** 10.1186/s13030-024-00320-w

**Published:** 2025-02-14

**Authors:** Maitreyi Redkar, Azizuddin Khan

**Affiliations:** Psychophysiology Laboratory, Department of Humanities and Social Sciences, Indian Institute of Technology Bombay, Mumbai, 400 076 India

**Keywords:** Focused attention, Divided attention, Accuracy, Reaction time, Endocrinal disturbances

## Abstract

**Background:**

Polycystic Ovary Syndrome (PCOS) is an endocrinal dysfunction characterized by androgen excess, irregular or absent menstruation, and polycystic ovarian morphology. While extensive research is conducted on the biochemical and medical ramifications of PCOS. However, there is not much research on cognitive mechanisms, especially attention. Attention is the fundamental cognitive ability that influences other cognitive and psychological phenomena. Therefore, the present study attempts to investigate the effect of PCOS on attention.

**Methods:**

Flanker’s task examining focussed attention and Posner’s cueing task measuring divided attention was administered to 173 female participants, of which 101 constituted the PCOS group and the remaining were control. The Analysis of Variance was used to analyze the data.

**Results:**

These findings demonstrated that the PCOS group took longer in focused attention, 557.21 milliseconds (SD = 169.70), compared to the reaction time of 462.88 milliseconds (SD = 120.80) in divided attention. Concerning accuracy, the PCOS group made more errors in the focused attention task at 0.98 (SD = 0.41), while for the divided attention task, it was 0.99 (SD = 0.27).

**Conclusions:**

Women with PCOS showed more error and slower reaction time in focused attention.

## Background

Polycystic Ovary Syndrome (PCOS) is an endocrinal dysfunction affecting women during the reproductive years [1, 2]. The diagnosis of PCOS includes any two out of the three following characteristics: oligo-anovulation, hyperandrogenism, and polycystic ovaries (Rotterdam Consensus, 2004). The disturbed metabolic and hormonal profile of PCOS increases the vulnerability to global cognitive deficits [3, 4]. Research indicates that women with PCOS have poorer performance across several cognitive domains such as memory [5], executive function [6, 7], verbal fluency [8], and visuospatial skills [9]. However, one of the important areas that play a crucial role before and during cognitive functioning is attention.

Attention is the allocation of limited cognitive processing resources [10], which supports a wide range of cognitive and behavioural functions. When functioning as a precursor to cognitive functioning, focused attention filters relevant from irrelevant environmental stimuli to ensure only essential sensory information reaches higher cognitive processes. This focused attention is required for everyday tasks such as reading and studying [11]. Additionally, divided attention enables individuals to manage multiple tasks in complex environments, such as balancing household and professional duties. This type of attention relies on alerting, orienting, and cognitive control to maintain and shift attention to the relevant stimuli.

Mehrabadi et al. [12] found poorer performance in attention among women with PCOS. However, the sample was recruited from the hospital, indicating that the significant medical distress and elevated levels of androgens may have affected their performance. Sukhapure et al. [9] reported that despite individualized treatment improving androgen-dependent psychiatric symptoms in PCOS women, poor attentional performance showed no significant improvement. Similarly, Showkath et al. [13] also identified subclinical attention impairments among PCOS women, supported by neurophysiological indicators of altered brain activity. Li et al. [7] linked elevated androgen levels and increased luteinizing hormonal levels to longer attentional response duration in PCOS women, though uncontrolled variables like social experience and occupation of the participants may have impacted these findings. Likewise, Franik et al. [14] found elevated androgens and insulin resistance levels associated with cognitive impairments, especially attention. However, the lack of a control group made it difficult for comparative analysis.

In contrast, despite increased androgen levels impairing cognitive performance, Sukhapure et al. [15] found no significant attentional differences between PCOS and non-PCOS groups. The study’s findings may be limited by the effects of psychotropic medications consumed by some PCOS participants and an increased risk of type 2 errors due to the extensive battery of cognitive tests. Rees et al. [4] reported no attentional differences despite white brain matter degradation related to elevated insulin and androgen levels. Similarly, Castellano et al. [6] observed no attentional disparities, even as insulin resistance affects brain glucose metabolism. Both studies faced limitations due to small sample sizes.

This ambiguity is highlighted in the meta-analysis by Perović et al. [3], which synthesized research on hormonal, biochemical, and metabolic links between PCOS and attention. However, these associations remain speculative, as the findings are largely based on extrapolations from related conditions like diabetes, obesity, and infertility rather than direct evidence from PCOS itself.

Additionally, Mehrabadi et al. [12, 13] have used the self-report Montreal Cognitive Assessment (MoCA) to evaluate attention along with other cognitive abilities. Other studies like Sukhapure et al. [6, 4, 9, 15] employed the Digit Span test, in addition to other tests assessing various cognitive abilities, including attention. Similarly, Franik et al. [14] examined attention along with other cognitive processes using experimental tasks such as Trail Making Test, Stroop Test, and Verbal Fluency Test, which are not appropriate tools for assessing attention. Li et al. [7] is the only study that administered the Attention Network Task (ANT) on PCOS participants, finding links between fluctuating androgen levels and brain network abnormalities affecting divided attention. The varied methodologies across studies make it difficult to understand which type of attentional abilities are most impacted by PCOS. As attention can be both a precursor to and a cognitive function, studying attention is crucial for understanding the cognitive performance of women with PCOS. Thus, this study evaluated the impact of PCOS on both focused and divided attention.

## Pilot study

A pilot study with six participants (M_age_ = 28.16 years; SD = 2.67) was conducted to refine experimental procedures before the main study for assessing attention in women with PCOS. There were three participants in the PCOS and three in the control group.

The study found that the PCOS group exhibited slower reaction times, as observed from the focused attention performance of the PCOS group (M_RT_ = 611.69, SD_RT_ = 13.07), while the control group responded faster at (M_RT_ = 493.27, SD_RT_ = 10.12). Similarly, for divided attention, the PCOS group showed a longer response time (M_RT_ = 690.14, SD_RT_ = 83.48) than the control group (M_RT_ = 414.25, SD_RT_ = 72.25).

The pilot study also found that the PCOS group had poorer accuracy on the task evaluating focused attention (M_Acc_ = 0.89, SD_Acc_ = 0.11), while the control group achieved high accuracy (M_Acc_ = 1, SD_Acc_ = 0). Similarly, the PCOS group had lower accuracy (M_Acc_ = 0.91, SD_Acc_ = 0.78) than the control group (M_Acc_ = 1, SD_Acc_ = 0).

The pilot study demonstrated that the PCOS group performed poorly in attention tasks, had slower response times, and made more errors than the control group. Since the pilot study was primarily conducted to remove any errors in the experimental conduction, this data was not considered for formal analysis.

## Methods

### Participants

A total of 173 females have participated in the study (M_age_ = 25.62; SD = 4.10). There were 101 participants diagnosed with PCOS as per the Rotterdam Criteria (M_age_ = 25.75; SD = 4.33; Ages from 19 to 35), and the control group consisted of 72 controls (M_age_ = 25.44; SD = 3.71; Ages from 19 to 33).

An a priori power analysis was conducted using G*Power version 3.1.9.7 [16] for sample size estimation. With a significance criterion of α = 0.05 and power = 0.80, the minimum sample size needed was *N* = 150 for ANOVA. Thus, the obtained sample size of *N* = 173 is more than adequate to test the study hypothesis.

The PCOS participants, recruited through support groups and medical practitioners, self-reported that their diagnosis was made by a qualified medical professional. The control group participants also self-reported not having PCOS. Both groups provided recent hormonal investigations (within the last year) to the researchers, who verified the presence or absence of the diagnosis by the consulting medical doctor before including the participants in the study. Additionally, all participants resided in Maharashtra, India, and could understand English instructions.

The participants with other endocrinal disorders, gynecological conditions such as pregnancy or menopause, psychiatric illness, and/or who were currently under any medication were excluded from the study. Participants with visual impairments that could affect their performance were also excluded from the study.

The demographic characteristics, participant numbers, and biochemical signs of PCOS and non-PCOS groups are shown in Table 1. Blood tests measuring participants’ hormonal levels were conducted four to six days after their menstrual cycle onset. These biochemical assessments were completed prior to their inclusion in the study. It can be observed that women with PCOS have lower levels of serum Follicle-Stimulating Hormone (FSH) and Estradiol, along with higher levels of total Testosterone. The variability in the hormone levels is greater in the PCOS group, indicating more hormonal fluctuations. However, no further analysis was performed on these biochemical characteristics beyond their initial evaluation for study eligibility.

**Table 1:** Demographic and biochemical characteristics of PCOS and non-PCOS participants

Characteristic	PCOS group	Non-PCOS group
N	101	72
Age (in years)	25.75 (SD = 4.31)	25.44 (SD = 3.79)
Weight (in Kgs)	76.32 (SD = 12.29)	69.58 (SD = 6.44)
Sr. FSH (in mIU/mL)	4.68 (SD = 7.68)	7.89 (SD = 4.91)
Sr. LH (in mIU/mL)	9.64 (SD = 2.56)	10.08 (SD = 3.18)
Sr. Estradiol (in pg/ml)	26.89 (SD = 13.92)	98.23 (SD = 4.36)
Sr. Total Testosterone (in ng/dL)	74.68 (SD = 26.74)	18.24 (SD = 4.81)

*All the serum concentrations were collected at the follicular level of ovulation

### Design

The study evaluated the impact of PCOS on the performance of attention tasks, specifically the accuracy and the speed at which the participants responded. The experimental design was a 2 (condition: PCOS vs. control) x 2 (attention: accuracy vs. reaction time) mixed factorial design. The between-group subjects included PCOS and the non-PCOS. The within-group subjects were the accuracy and reaction time of attention performance.

The data from the participants were analysed using the Analysis of Variance abbreviated to ANOVA. This statistical method compares the means of different groups (PCOS vs. control) in terms of their performance on attention tasks (focused and divided attention). As there are two levels for both types of attention tasks (reaction time and accuracy), it allows for comparing all the means simultaneously, accounting for both within-group and between-group variations.

### Measures and procedures

#### Focussed attention (Flanker task)

The Eriksen Flanker task was designed to detect the interference of the ‘noise letters’ or distractors on the main task [17]. In the present study, pictorial nonverbal stimuli of animated fish were employed. The nonverbal stimuli eliminated any biases of the English speakers.

The participants were provided with the following instructions - “In the first task, you have a row of fish provided to you. You will be required to be attentive to the fish in the middle of the combination set. In case you see the fish swimming towards the left, press the alphabet key ‘N’; if you see the fish swimming towards the right, press the alphabet key “M’. Once you respond, you will be given feedback on whether you are right (plus sign turns into a green tick mark) or wrong (plus sign turns into a red cross mark).”

#### Divided attention (Posner Cueing task)

The Posner Cueing task measures divided attention by assessing the individuals’ ability to distinguish between informative and uninformative cues by shifting attention [18]. In this study, the primary analysis for divided attention included only endogenous cueing of an ambiguous nature (the cue did not indicate the side of the stimuli appearing).

The participant was provided with the following instructions - “Now, in the second task, you will be shown two boxes. Then, after seeing a plus sign, a green circle will appear in either of those boxes, which is to be ignored. Then, the stimuli will be provided. If you see the thumbs-up sign in the left box, press the alphabet key ‘M’; if you see the thumbs-up sign in the right box, press the key ‘N’. Once you respond, you will be given feedback only when you are wrong.”

The experiment was conducted in a quiet laboratory set-up room. Both tasks were designed and executed through the Gorilla Experiment Builder. For both measures of attention, participants attempted 20 practice trials before the 200 trials of the main experiment. The total duration of the experiment lasted for 10 min.

The study was in adherence to the Declaration of Helsinki, wherein a consent form and a demographic questionnaire were provided to each participant. The objective of the study and the relevance of their contribution were explained before the experiment. They were assured of ethical approval from the Institute Ethics Committee (IITB-IEC/2022/016).

## Results

The study investigated the impact of PCOS on attention. Two tasks of focused and divided attention were administered to PCOS and control participants. The performance of the tasks was analysed based on accuracy and reaction time.

**Fig. 1:**
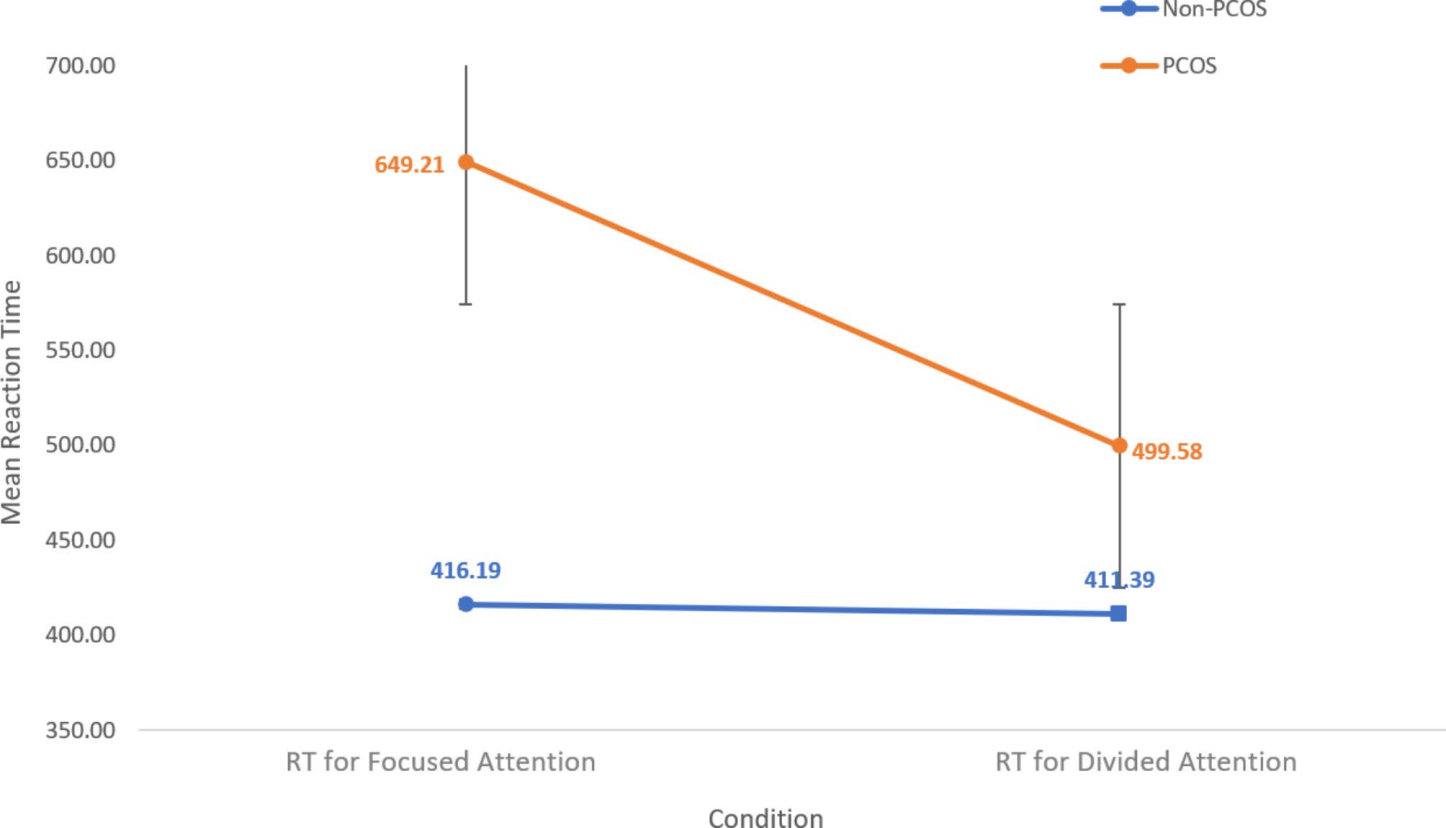
Difference in the reaction time of PCOS and non-PCOS

The descriptive statistics of the RT are presented in Fig. 1, wherein the blue line represents the performance of the non-PCOS control group, while the red line illustrates the performance of the PCOS participants.

The overall reaction time of the total sample for focussed attention tasks was found to be (M_RT_ = 512.16, SD = 168.36) and for divided attention tasks (M_RT_ = 463.09, SD = 118.68). Observing the reaction time for the focused attention tasks, it was found that the PCOS group took longer (M_RT_ = 649.18, SD = 171.28), while the control group responded faster (M_RT_ = 416.19, SD = 71.95). Similarly, in the divided attention task, the PCOS group took longer to respond (M_RT_ = 499.58, SD = 129.29), and the control took a shorter time to respond (M_RT_ = 411.39, SD = 76.15).

**Fig. 2:**
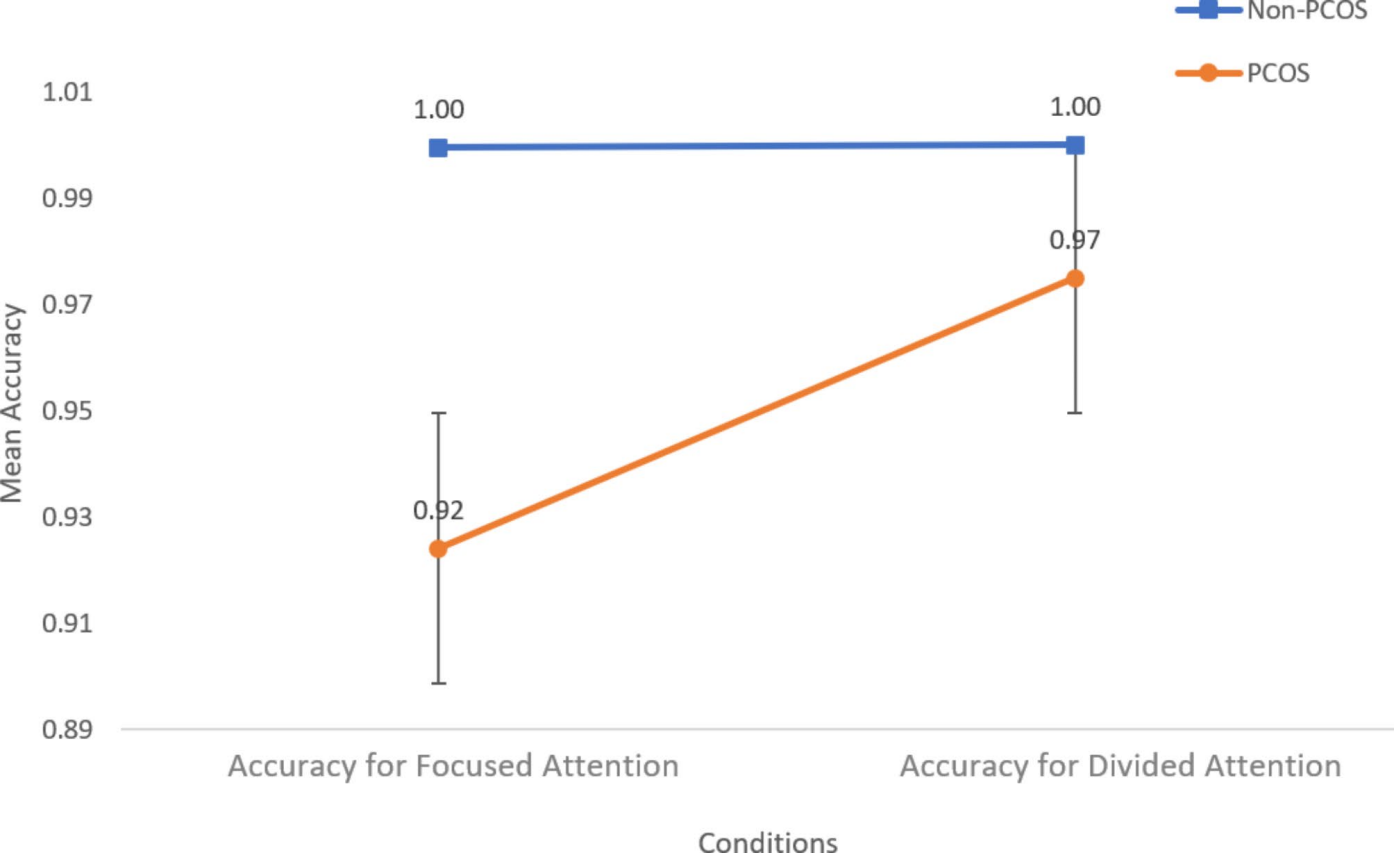
Difference in accuracy among PCOS and non-PCOS

Additionally, Fig. 2 presents the descriptive statistics for accuracy, with the blue line representing the performance of the non-PCOS control group and the red line indicating the performance of the PCOS participants.

The overall accuracy of the total sample for focussed attention tasks was found to be (M_Acc_= 0.95, SD = 0.11) and for divided attention tasks (M_Acc_= 0.99, SD = 0.04). Observing the accuracy for the focused attention tasks, it was found that the PCOS group made more errors (M_Acc_= 0.92, SD = 0.14), while the control group had higher accuracy (M_Acc_= 0.99, SD = 0.003). Similarly, in the divided attention task, the PCOS group had poorer accuracy (M_Acc_= 0.97, SD = 0.05), and the control made no errors (M_Acc_= 1, SD = 0).

The analysis of ANOVA between groups revealed that the main effect of the condition (PCOS vs. control) was significant at (*F* (1,171) = 152.73, *p* = 0.001, η_p_^2^ = 0.472). For the within-subjects, the main effect of accuracy was significant for attention at (*F* (1,171) = 40.58, *p* = 0.001, η_p_^2^ = 0.192). Moreover, the interaction effect for accuracy – conditions was significant at, (*F* (1,172) = 35.69, *p* = 0.001, η_p_^2^ = 0.173). For the within-subjects, the main effect of reaction time was also significant for focussed attention at (*F* (1,171) = 40.57, *p* = 0.001, η_p_^2^ = 0.192), and the interaction effect for RT – conditions was significant at (*F* (1,172) = 35.68, *p* = 0.001, η_p_^2^ = 0.173). Similarly, the interaction effects between accuracy – RT – condition were significant *(F* (1,171) = 141.19, *p* = 0.001, η_p_^2^ = 0.452). This is detailed in Table 2, attached below.

**Table 2:** The summary of analysis of variance for attention tasks (focussed attention vs. divided attention) on Condition (PCOS vs. Control)

Source	df	Mean Square	F-value	Sig.	η_*p*_^2^
**Between subjects**					
Condition	1	2281910.09	152.73	0.001	0.472
Error	171	14940.70			
**Within subjects**					
Accuracy	1	250518.40	40.58	0.001	0.192
Accuracy * Condition	1	20347.70	35.70	0.001	0.173
Error (Accuracy)	171	6173.47			
RT	1	250545.05	40.57	0.001	0.192
RT * Condition	1	220328.73	35.68	0.001	0.173
Error (RT)	171	6175.29			
Accuracy * RT * Condition	1	1084223.12	141.19	0.001	0.452
Error (Accuracy * RT)	171	7679.240			

η_p_^2^ -Partial Eta Square

## Discussion

The present study investigated the impact of PCOS on focused and divided attention using Flanker and Posner’s cueing task. The study’s findings revealed a significant difference between the performance (accuracy and RT) between the PCOS and control group. The result showed a significant interaction effect between accuracy and condition. It indicates that the PCOS participants made more errors and took longer to respond to attention tasks than control. It was observed that focussed attention was more impacted than divided attention among women with PCOS.

Focused attention typically involves concentrating on a single stimulus, while divided attention requires the simultaneous processing of multiple stimuli. According to Broadbent’s Filter Model of Attention (1957), attention is a precursor to cognitive functioning as it filters relevant from irrelevant environmental stimuli to ensure that only the most pertinent sensory input reaches higher cognitive processes. This focused attention is required for everyday tasks such as reading and studying.

One possible reason for the attentional disruptions observed in women with PCOS may be the hormonal imbalances that affect neurotransmitter systems and brain function (CITATION). These imbalances may impair the ability to filter sensory information, causing higher distraction, reduced accuracy, and slower response times. The findings of this study are consistent with research by Li et al. [7], which showed that elevated testosterone levels in women with PCOS result in sub-optimal brain activity during attention tasks, leading to poor performance. Similarly, Franik et al. [14] observed that increased androgens, higher cortisol, and lower progesterone levels in women with PCOS negatively impacted attention task performance. Barnard et al. (2007) also found that hormonal imbalances contribute to slower reaction times. Despite Mehrabadi et al. [12] reporting lower attention scores in women with PCOS, the study did not find a direct correlation with hormonal imbalances. They attributed the deficits instead to oxidative stress and mental distress related to severe acne, mentioning a potential bias due to the small sample size.

Another factor affecting focused attention in PCOS may arise from the metabolic conditions of insulin resistance. This condition impairs glucose metabolism and neural processing, leading to difficulty in filtering out irrelevant stimuli, potentially reducing processing speeds and attentional performance. Studies by Castellano et al. [6] and Franik et al. (2023) support this, demonstrating that impaired glucose metabolism and insulin resistance interferes with brain activity, causing slower speeds and more errors in attention tasks.

As observed above, PCOS experiences issues with not just focused attention but also divided attention. Thus, the Theory of Attention by Posner and Petersen [19] states that divided attention involves the interaction of alerting, orienting, and cognitive control to maintain focus and shift attention to relevant stimuli to manage cognitive resources efficiently.

One potential reason for poor attentional deficits could be the impairment in the orientation ability caused by hormonal imbalances in women with PCOS. This results in a longer time to identify and process relevant information due to difficulties in filtering out irrelevant stimuli. Showkath et al. [13] found that poor neural processing, as indicated by reduced alpha and increased theta activity, was associated with slower response times and difficulty attending to the relevant stimuli. Similarly, Soleman et al. [20] observed androgen-dependent altered brain activity in attention-related areas among PCOS women, leading to slower and less accurate performance. Rees et al. (2015) noted that hormonal imbalances could erode brain white matter, potentially impairing attention-related areas.

Women with PCOS may struggle with divided attention tasks due to impaired cognitive control, potentially stemming from the mental fatigue associated with PCOS. Posner’s Theory of Attention (1990) highlights the importance of cognitive control in processing relevant stimuli. However, mental fatigue, characterized by anxiety and frustration, makes it more challenging for women with PCOS to focus on relevant information [21, 22]. Ananthasubramanian et al. [22] support this argument by demonstrating the contribution of neurotransmitter impairments to emotional fatigue causing high distractibility.

As PCOS may impair cognitive control and subsequently affect attention, it is also likely that impairments in other cognitive abilities, such as working memory, contribute to attentional difficulties. Working memory is critical for holding and manipulating short-term information, essential for filtering irrelevant stimuli and shifting attention between tasks [24]. Therefore, any deficits in working memory could lead to challenges in cognitive control, resulting in poorer attentional performance.

In the present study, women with PCOS demonstrated lower accuracy in divided attention tasks, which may be attributed to the negative impact of PCOS on neuroanatomy and neurotransmitter function, affecting working memory. Previous research supports this, with Li et al. [7] finding that women with PCOS exhibit longer response times and lower accuracy on tasks involving working memory, attention, and executive function, further highlighting the interconnected nature of these cognitive abilities. Even Schattman & Sherwin (2007) revealed that women with PCOS were performing poorer than the non-PCOS on working memory.

However, the present study had several limitations such as participants diagnosed with PCOS were treated as a homogeneous experimental group without further investigation into the specifications of their phenotypes. Additionally, while efforts were made only to recruit participants who were not consuming medications, the study relied on self-reported data. If participants did not accurately disclose their medication use, this could have influenced the study’s results. Another limitation of this study was the exclusion of controlling for the psychosocial background of the participants. The participants came from different socio-economic backgrounds, which may have affected their performance.

Future research can consider the different age cohorts to observe potential variations in their cognitive performance. Employing diverse psychophysiological methods, such as EEG or eye tracking, would contribute to a more comprehensive understanding of the impact of PCOS on attention.

## Conclusion

The study highlighted the impact of PCOS on attention. Attention is required to process any stimuli from the environment. The findings suggest that poor accuracy and slow reaction time are observed in attention tasks, especially focussed attention, and this is the possible reason why many other studies investigating cognition have found an impacted performance in PCOS.

## Data Availability

The datasets generated during and/or analysed during the current study are available from the corresponding author upon reasonable request.
